# The integrative bioinformatic analysis deciphers the predicted molecular target gene and pathway from curcumin derivative CCA-1.1 against triple-negative breast cancer (TNBC)

**DOI:** 10.1186/s43046-021-00077-1

**Published:** 2021-08-02

**Authors:** Dhania Novitasari, Riris Istighfari Jenie, Jun-ya Kato, Edy Meiyanto

**Affiliations:** 1 Doctoral Student in the Faculty of Pharmacy, Universitas Gadjah Mada, Yogyakarta, 55281 Indonesia; 2 Cancer Chemoprevention Research Center, Faculty of Pharmacy, Universitas Gadjah Mada, Yogyakarta, 55281 Indonesia; 3 Macromolecular Engineering Laboratory, Department of Pharmaceutical Chemistry, Faculty of Pharmacy, Universitas Gadjah Mada, Sekip Utara, Yogyakarta, 55281 Indonesia; 4 Laboratory of Tumor Cell Biology, Nara Institute of Science and Technology, Ikoma, Nara 630-0192 Japan

**Keywords:** TNBC, CCA-1.1, Bioinformatics, Cell cycle, Mitosis

## Abstract

**Background:**

The poor outcomes from triple-negative breast cancer (TNBC) therapy are mainly because of TNBC cells’ heterogeneity, and chemotherapy is the current approach in TNBC treatment. A previous study reported that CCA-1.1, the alcohol-derivative from monocarbonyl PGV-1, exhibits anticancer activities against several cancer cells, as well as in TNBC. This time, we utilized an integrative bioinformatics approach to identify potential biomarkers and molecular mechanisms of CCA-1.1 in inhibiting proliferation in TNBC cells.

**Methods:**

Genomics data expression were collected through UALCAN, derived initially from TCGA-BRCA data, and selected for TNBC-only cases. We predict CCA-1.1 potential targets using SMILES-based similarity functions across six public web tools (BindingDB, DINIES, Swiss Target Prediction, Polypharmacology browser/PPB, Similarity Ensemble Approach/SEA, and TargetNet). The overlapping genes between the CCA-1.1 target and TNBC (CPTGs) were selected and used in further assessment. Gene ontology (GO) enrichment and the Kyoto Encyclopedia of Genes and Genomes (KEGG) network analysis were generated in WebGestalt. The protein–protein interaction (PPI) network was established in STRING-DB, and then the hub-genes were defined through Cytoscape. The hub-gene’s survival analysis was processed via CTGS web tools using TCGA database.

**Results:**

KEGG pathway analysis pointed to cell cycle process which enriched in CCA-1.1 potential targets. We also identified nine CPTGs that are responsible in mitosis, including *AURKB*, *PLK1*, *CDK1*, *TPX2*, *AURKA*, *KIF11*, *CDC7*, *CHEK1*, and *CDC25B*.

**Conclusion:**

We suggested CCA-1.1 possibly regulated cell cycle process during mitosis, which led to cell death. These findings needed to be investigated through experimental studies to reinforce scientific data of CCA-1.1 therapy against TNBC.

**Supplementary Information:**

The online version contains supplementary material available at 10.1186/s43046-021-00077-1.

## Background

Triple-negative breast cancer (TNBC) subtypes contribute around 20% of total diagnosed breast cancer cases [1]. The research related to TNBC is challenging since, despite extensive existing chemotherapy agents available in the market, the patients’ survival rate remains low due to chemoresistance, relapse, and even metastasis, worsening the prognosis. The heterogeneity in TNBC cells makes the variance of biological behavior and evokes many researchers to find prospective chemotherapeutic drugs to overcome the aggressiveness of metastatic TNBC [2]. To date, many genomic and transcriptomic data from cancer patients had been publicly deposited through databases (i.e., The Cancer Genome Atlas or TCGA), and they are available to use for projecting the predicted molecular mechanism with different approaches.

**Fig. 1:**
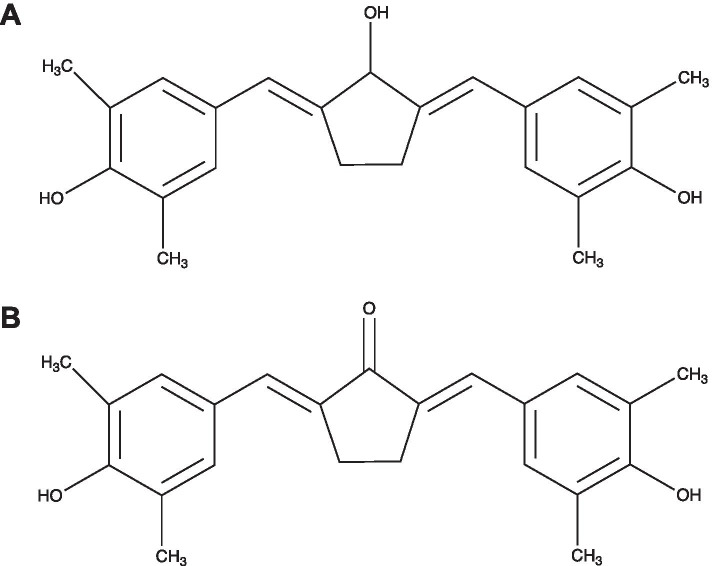
Chemical structure of (**A**) CCA-1.1 and (**B**) PGV-1

Many studies reveal curcumin and its derivatives promote succeeding potential as candidate chemotherapy with multi targets against breast cancer. This time, we focused on the CCA-1.1 compound, a curcumin derivative from PGV-1 (Fig. 1) which demonstrated anticancer activities through in vitro experiments in leukemic, colorectal, and breast cancer [3–5]. Notably, in murine TNBC 4T1 cells, CCA-1.1 induced mitotic arrest and enhanced ROS level led to cellular senescence [3]. Furthermore, CCA-1.1 also worked synergistically with conventional chemotherapy doxorubicin to delay cell division and inhibited migration in metastatic breast cancer cells. Likewise, the molecular docking analysis of CCA-1.1 also explored several ROS scavengers (unpublished data). That information exhibits the potential development of CCA-1.1 for further investigation regarding its molecular pathway in cancer cells. A previous study documented that CCA-1.1 identified the putative targets in colon cancer, including *ERBB2*, *TP53*, and *MAPK1*, using bioinformatic analyses [6]. Therefore, this time, we determine the potential therapeutic targets for CCA-1.1 toward TNBC regulatory genes.

This study provided an integrative bioinformatics viewpoint to discover potential new targets and visualize cellular mechanisms of CCA-1.1 in TNBC. The predicted target gene of CCA-1.1 was collected from public online databases using the SMILES code of CCA-1.1. Simultaneously, the genomic data of TNBC patients were generated via UALCAN web portal using the TCGA-BRCA database. Both associated genes were made into a Venn diagram to visualize the intersection representing CCA-1.1 potential target genes (CPTGs). PPI network, KEGG pathway, GO enrichment, and survival analysis of top CPTGs hub genes demonstrate the clear and molecular path of CCA-1.1 in inhibiting TNBC progression. This present finding could be set as the basis for further developing CCA-1.1 for future multitargeted chemotherapy drugs for TNBC therapy (Fig. 1).

## Methods

### Data acquisition from genes expressed in TNBC

We collected the list of upregulated and downregulated genes within TNBC patients from UALCAN (http://ualcan.path.uab.edu/) and select the data to manifest retrieved from The Cancer Genome Atlas (TCGA) breast cancer [7]. The top 250 genes from each category were downloaded for further analysis.

### The determination of putative CCA-1.1 targets

We used several web tools to acquire the potential target gene for CCA-1.1, such as BindingDB (http://www.bindingdb.org/bind/index.jsp) [8], DINIES (https://www.genome.jp/tools/dinies/) [9], Polypharmacology browser or PPB (http://gdb.unibe.ch/) [10], Similarity ensemble approach or SEA (https://sea.bkslab.org/) [11], SwissTargetPrediction (http://www.swisstargetprediction.ch/) [12], and TargetNet (http://targetnet.scbdd.com/) [13]. We used the MarvinJS feature from ChemAxon (https://chemaxon.com/products/marvin-js) to draw the chemical structure and retrieve the SMILES code be inputted into the databases. All the settings in each database were selected as default. After removing the duplication of target genes, we used Vienny 2.1 (https://bioinfogp.cnb.csic.es/tools/venny/) to determine the overlapping genes between significant genes in TNBC and CCA-1.1 target genes. We classified each gene based on their protein class through PANTHER v.16 (http://www.pantherdb.org/).

### Functional annotation chart and pathway enrichment analysis

We used the Kyoto Encyclopedia of Genes and Genomes (KEGG) pathway and Gene Ontology (GO) databases to analyze the enrichment of the overlapping genes. The enrichment of the KEGG pathway and GO analysis were processed through Overrepresentation Enrichment Analysis (ORA) via WebGestalt 2019 (http://www.webgestalt.org/) [14] and chose false discovery rate (FDR) less than 0.05 as the cut-off value. We then visualized the graph using GraphPad v.7.

### Construction of the PPI network of the overlapping genes

The PPI network analysis was constructed with STRING-DB 11.0b [15] with confidence scores greater than 0.7. Then, the network analysis was generated through the latest Cytoscape [16]. According to degrees, we ranked the selected genes and chose the top 10 genes analyzed in CytoHubba. We considered the ten highest-ranked genes for further assessment.

### The survival analysis of associated genes in TNBC patients

We curated the survival analysis categorized by overall survival and disease-free survival for each gene via cancer target gene screening (http://ctgs.biohackers.net/) [17] and selected TCGA breast cancer data year 2018, then filtered based on subtype TNBC. The data were visualized into a Kaplan–Meier plot. The plot consists of separate patients divided into high and low expression groups based on the gene transcriptional expression level of a given gene, the hazard ratio (HR) with the 95% confidence interval, and the log-rank *P* value were calculated and displayed on the chart.

## Results

### Data collection and processing of DEGs in TNBC and CCA-1.1 predicted target genes

**Fig. 2:**
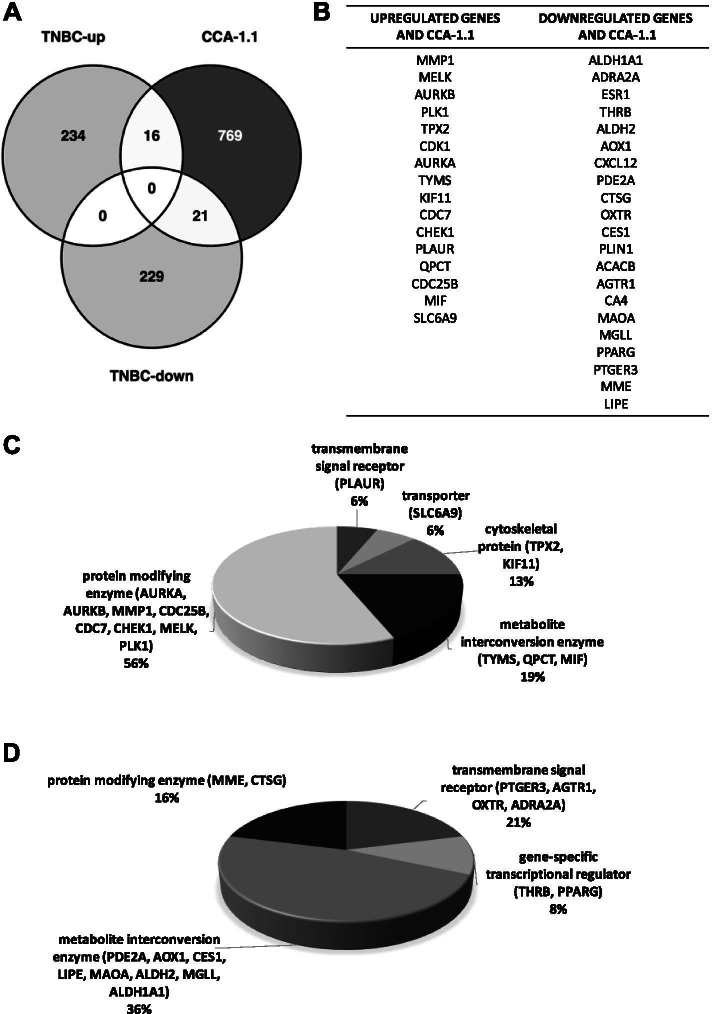
Several CCA-1.1 target genes overlapped with TNBC biomarkers from The CGA database, according to Venn diagram (**A**), resulted in a total of 37 potential therapeutic targets of CCA-1.1 (CPTGs) as listed in table (**B**). The classification of genes based on their protein class using PANTHER for the upregulated genes (**C**) and downregulated genes (D) overlapped with CCA-1.1

We screened out a total of 250 upregulated and downregulated genes that presented in TNBC patients according to the TCGA breast cancer project (Supplementary Data 1), and later we called it as differentially expressed genes (DEGs) of TNBC. Some of the genes were noticeable to be responsible for breast cancer progression. Next, we processed the predicted target gene of CCA-1.1 using six different online databases to get more comprehensive data from BindingDB, DINIES, SEA, SwissTargetPrediction, TargetNet, and poly-pharmacology browser. A total of 806 genes (without duplicated genes) from the databases were encoded as CCA-1.1 target genes. Furthermore, we used the Venn diagram to cross the genes between the CCA-1.1 target and DEGs of TNBC. We found 16 overexpressed genes and 21 downregulated genes were listed as the overlapping genes to be used for further analysis as CCA-1.1 potential target genes or CPTGs (Fig. 2A and 2B). Next, we categorized the overlapping genes according to protein class with the help of PANTHER web tools. Most of the listed genes were classified into protein modifying enzymes, with 56% in upregulated genes and 36% in downregulated genes, respectively (Fig. 2C and D). Looking at this information, we decided to focus on the overlapping genes associated with overexpressed genes in TNBC for further investigation.

### GO and KEGG pathway enrichment of CPTGs

**Fig. 3:**
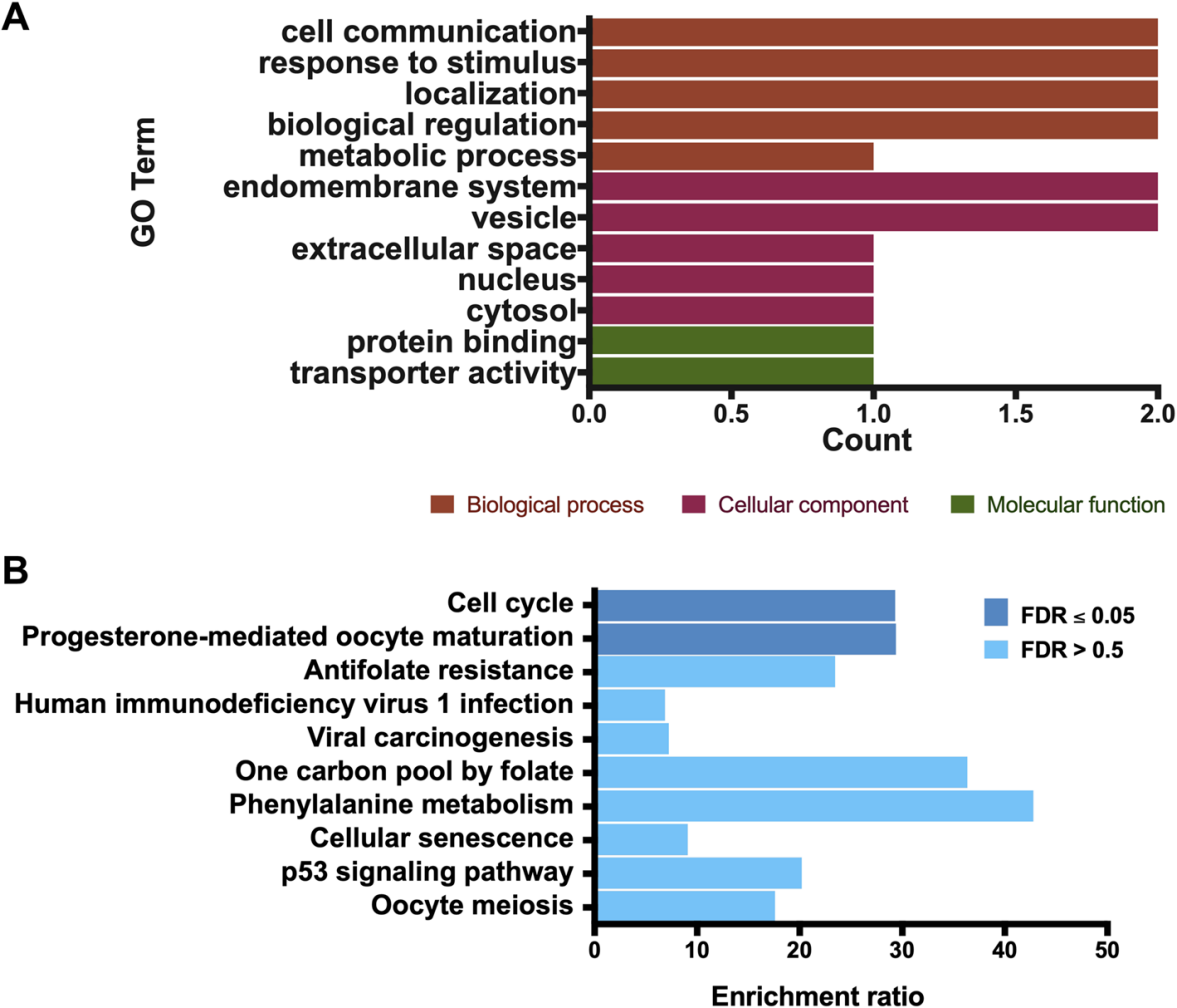
(**A**) The involvement of CPTGs in biological process (red bar), molecular function (blue bar), and cellular component (green bar) using gene ontology (GO) database. (**B**) Gene enrichment analysis from CPTGs using KEGG pathway which processed through Webgestalt

We expound on the functional annotations of CPTGs through GO function and KEGG pathway enrichment analysis using Webgestalt web tools. We also categorized the GO functional with over-representation analysis (ORA) and revealed that CPTGs are involved in cell communication, localization, and metabolic process (Fig. 3A, brown bar). Besides, CPTGs were related to protein binding and transporter activity (Fig. 3A, green bar) located in the endomembrane system, nucleus, and cytosol (Fig. 3A, pink bar). Moreover, the CPTGs were enriched in the cell cycle based on KEGG pathway analysis, as shown in Fig. 3B. Given this information, we later focused on exploring genes involved in cell cycle regulation.

### PPI network and hub-genes analysis of CPTGs

**Fig. 4:**
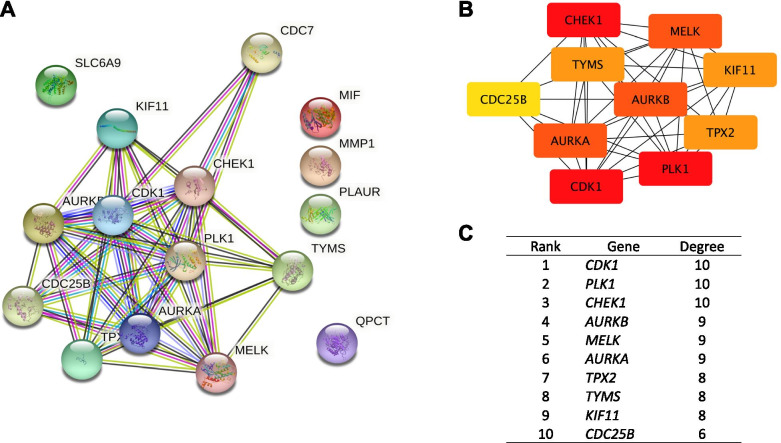
(**A**) The protein–protein interaction of CPTGs established in STRING. (**B**) The top ten genes of CPTGs were selected based on the highest degree score visualized in Cytohubba in Cytoscape. (**C**) The rank list from the top ten CPTGs genes

We curated the CPTGs to determine each gene’s interactions (also with other interactors) and visualized the network through STRING web tools. We found 16 nodes with 45 edges and an average node degree of 5.62 displayed in the PPI network (Fig. 4A). We ranked the nodes according to the node’s degree and listed the top ten genes with the highest score (Fig. 4B and C). With the help of the molecular signature database (MSigDB) (https://www.gsea-msigdb.org/gsea/msigdb/index.jsp), we found that nine genes from 16 overexpressed genes are included in G2/M checkpoint genes, as listed in Table 1. We suggested that CCA-1.1 potentially targets cell cycle regulation in TNBC.

**Table 1:** The CPTGs which involved in the G2/M checkpoint hallmark according to MSigDB

Gene	Protein name	Role in cell cycle regulation
AURKB	Aurora kinase B	Maintains kinetochore-microtubule association in metaphase and relocalizes to the midbody of the cell from anaphase to telophase
PLK1	Polo-like kinase 1	Regulates centrosome disjunction, activates of cyclin and cyclin-dependent kinases (CDKs), spindle assembly, and chromosome separation
TPX2	Targeting protein for Xklp2	Forms the mitotic spindle by activates the Aurora kinase A
CDK1	Cyclin-dependent kinase 1	Promotes cell cycle progression from G2 to mitosis by generating chromosome condensation and microtubule dynamics
AURKA	Aurora kinase A	Favors G2 to mitosis transitioning and contributes to centrosome separation
KIF11	Kinesin family member 11	Intercedes centrosome segregation and conformation of the bipolar mitotic spindle
CDC7	Cell division cycle 7-kinase	Stimulates Aurora kinase B activity during mitosis progression
CHEK1	Checkpoint kinase 1	Relieves cyclin B/Cdk1 in the nucleus during mitosis, targets spindle assembly checkpoint proteins
CDC25B	M-phase inducer phosphatase 2	Promotes progression from G2 to mitosis by activating Cdk1/cyclin B complex for centrosome microtubule nucleation, phosphorylates Aurora-A to permit entry into mitosis by giving the first stimulus of Cdk1 activity

### Survival analysis of CPTGs in triple negative breast cancer patients

**Fig. 5:**
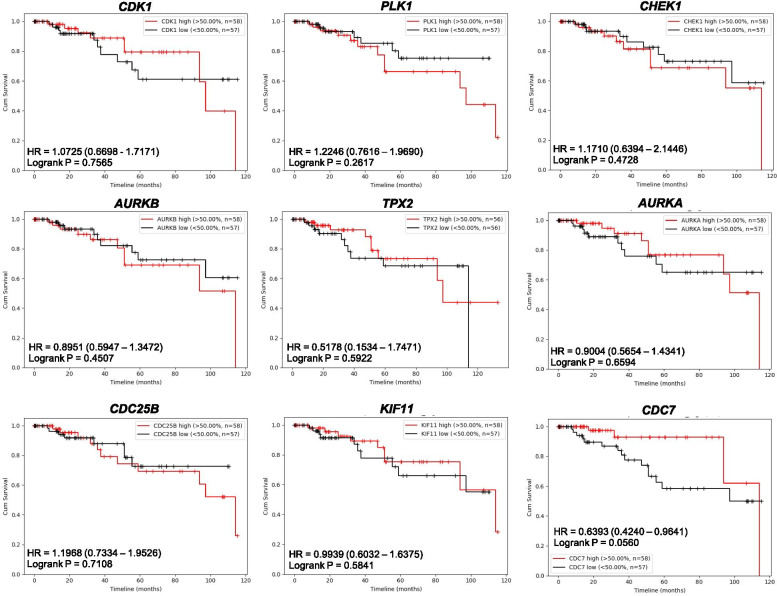
The overall survival analysis from *AURKB*, *PLK1*, *CDK1*, *TPX2*, *AURKA*, *KIF11*, *CDC7*, *CHEK1*, and *CDC25B* genes in triple negative breast cancer patients

**Fig. 6:**
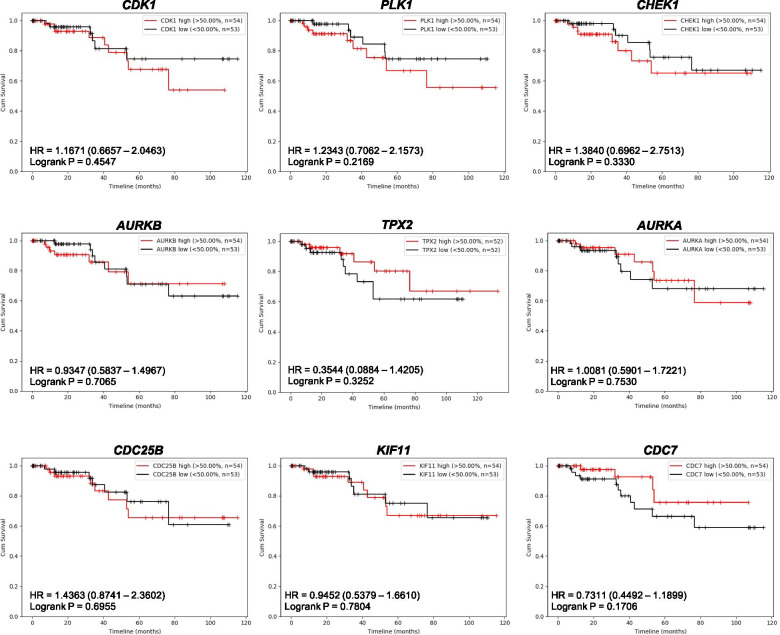
The disease-free survival analysis from *AURKB*, *PLK1*, *CDK1*, *TPX2*, *AURKA*, *KIF11*, *CDC7*, *CHEK1*, and *CDC25B* genes in triple negative breast cancer patients

Using the CTGS web tools that provided the TCGA-breast cancer data project, we analyzed the survival analysis using two parameters: overall survival (OS) and disease-free survival (DFS) with median cut-off. We plotted it into a Kaplan–Meier graph for the CPTGs responsible for the G2/M checkpoint (*AURKB*, *PLK1*, *CDK1*, *TPX2*, *AURKA*, *KIF11*, *CDC7*, *CHEK1*, and *CDC25B*). In a total of 115 TNBC patients, high expression of those respective genes generally decrease the overall survival (OS) during 110 months span, though the log-rank tests did not show significant prognostic value from those genes for overall survival (Fig. 5). Although statistically, there was no significant difference between high expressed and low expressed genes in patients, TNBC patients who had increased *CDK1*, *PLK1*, and *AURKA* intended to have a higher risk of relapse than patients with lower expression. Meanwhile, lower expression of *TPX2*, *CDC7*, and *AURKB* genes had poorer disease-free survival in TNBC patients (Fig. 6).

## Discussion

The current study attempted to identify the potential therapeutic targets of a novel curcumin analog, CCA-1.1, toward triple negative breast cancer (TNBC). Experimentally, CCA-1.1 performed a cytotoxic effect against murine-derived TNBC cell line, 4T1 cells. Moreover, CCA-1.1 inhibited cell cycle progression in mitosis, enhanced high intracellular reactive oxygen species (ROS) level, leading to senescence [3]. Given the preliminary in vitro results, we investigated the possible target genes associated with CCA-1.1 as a potential antineoplastic agent against TNBC. We used a public TCGA data network and selected the samples classified as TNBC to obtain better comprehensive genes that represented more critical in TNBC tumor. Through the UALCAN portal web, we collected each 250 lists of upregulated and downregulated genes in TNBC patients for further analysis. As for the CCA-1.1 potential target, we generated several online web tools through chemogenomic approaches using SMILE-based similarity, since computational drug-target interactions (DTIs) deemed much advantageous to elucidate the potential molecular pathway and also the possible target of our compounds. After TNBC genes and CCA-1.1 potential targets were processed, it resulted in 16 overexpressed genes and 21 low-expressed gene representing CCA-1.1 potential target genes (CPTGs). Since the overexpressed genes differ tumor cells to normal cells, we focused on the overexpressed genes that potentially targeted by CCA-1.1 in further analysis.

We highlighted that CPTGs are overrepresented in cell cycle based on KEGG pathway enrichment analysis. The dysregulation of the cell cycle enables uncontrollable cell multiplication; thus, the phenomenon marked as part of hallmark of cancer. During cell cycle, tumor cells annulled checkpoints to permit boundless division despite of aneuploidy and cellular deformity that would avoid non-cancer cells from multiplying. This phenomenon is attained by means of the accession of numerous genetic and epigenetic molecular adjustments that modulate key role of the cell cycle, and compel certain cellular dependencies in tumor cells to experience abnormal division [18]. Each subtype bears different molecular alteration. For instance, various reports revealed that TNBC cells display reliance on the spindle assembly checkpoint (SAC), arise expression of checkpoint genes in mitosis, and DNA damage response genes, apparently due to their high levels of genomic instability [19]. This subtype is also likely to present highly aneuploid cells with loss of TP53 function, thus, enhance the aggressiveness tumor growth and affect poor survival prognosis [20].

Among the 16 genes, nine of them are embroiled in cell cycle progression, particularly in G2/M phase. Moreover, some of listed genes are involved with each other for progression of mitosis. Aurora kinase A (AurA) phosphorylates PLK1 to drive centrosome maturation and involve in spindle poles and kinetochore. Moreover, the phosphorylated PLK1 activates CDK1 to permit mitosis entry and release AurA for binding with other mitotic proteins [21]. Upon mitotic entry, TPX2 also activates AurA for microtubule nucleation, which underlies in spindle assembly pathway [22]. Furthermore, the activation of Aurora kinase B (AurB) that possibly mediated through CHK1 cause phosphorylation of multiple substrates (including PLK1) to ensure chromosome segregation [23]. In other report, TPX2 stimulates KIF11 during spindle pole separation [24]. Considering the complex process during mitosis in cell cycle, targeting mitosis becomes advantageous for anticancer drugs.

Mitotic cascade is indeed a complex process and involve many unique proteins that regulate the progression in cell division. Prior study using leukemic cells demonstrated that PGV-1 inhibited cell cycle in prometaphase [25], the second stage in mitosis that begin when the nuclear envelope disassemble, thus, allow chromosomes into contact with microtubules arising from the two poles of the establishing mitotic spindle. During prometaphase, to make sure the chromosome attachment to spindle is secured, cells need to pass through spindle assembly checkpoint (SAC) which targets anaphase promoting complex/cyclosome (APC/C) [26]. The inactivation of the SAC due to CDK1 inhibition on APC and AurB activation leads a catastrophic, untimely entry into anaphase, regardless of the chromosome juxtaposition status. This creates to an uneven distribution of chromatids and genetic disproportion among daughter cells known as aneuploidy [27]. We presume that CCA-1.1 has involvement in those protein which cause centrosome formation failure, unlike the other antimitotic drugs (i.e., Taxol and Vinca alkaloids) whose targeted in microtubule, and this effect somehow affected mature neurite formation [28, 29]. These bioinformatic data delivers some valuable information to find the exact mechanism of these curcumin analogs which differ with existing antimitotic drugs.

We realized that experimental studies should support all these comprehensive bioinformatic studies to prove the therapeutic target(s) for CCA-1.1 in TNBC. Our results presented here give some insightful knowledge to explore the possible cellular mechanism of CCA-1.1 to kill TNBC cells. Alike its parent compound (PGV-1) [30], CCA-1.1 treatment-induced cell accumulation to arrest during mitosis phase. Given the result from our current bioinformatic study here, we suggest that CCA-1.1 may also have wide chances to target cell cycle process. Therefore, future studies focusing on metabolic reprogramming of CCA-1.1 in TNBC should be beneficial to be investigated to construct the molecular mechanism of CCA-1.1 as a prospective chemotherapy agent, notably for TNBC therapy.

## Conclusions

Altogether, using thorough bioinformatic approaches, we predict that CCA-1.1 has potential anticancer activities that mediated through mitosis in triple negative breast cancer.

## Supplementary Information

**Additional file 1: Supplementary Data 1**. Top 250 downregulated genes in TNBC based on TCGA-BRCA.

## Data Availability

The authors confirm that the data supporting the findings of this study are available within the article.

## Abbreviations

CCA-1.1: Cancer Chemoprevention Analog 1.1
TNBC: Triple negative breast cancer
PGV-1: Pentagamavunone-1
TCGA: The Cancer Genome Atlas
BRCA: Breast invasive carcinoma
SMILES: Simplified Molecular Input Line Entry System
DINIES: Drug-target interaction network inference engine based on supervised analysis
PPB: Polypharmacology browser
SEA: Similarity ensemble approach
GO: Gene Ontology
KEGG: Kyoto Encyclopedia of Genes and Genomes
CTGS: Cancer target gene screening
PPI: Protein-protein interaction
STRING-DB: Search Tool for the Retrieval of Interacting Genes/Proteins Database
AURKB: Aurora kinase B
PLK1: Polo-like kinase 1
CDK1: Cyclin dependent kinase 1
TPX2: Targeting protein for Xklp2
AURKA: Aurora kinase A
KIF11: Kinesin family member 11
CDC7: Cell division cycle 7
CHEK1: Checkpoint kinase 1
CDC25B: Cell division cycle 25B
ROS: Reactive oxygen species
ERBB2: Erb-B2 receptor tyrosine kinase 2
TP53: Tumor protein P53
MAPK1: Mitogen-activated protein kinase 1
CPTGs: CCA-1.1 potential target genes
ORA: Overrepresentation enrichment analysis
HR: Hazard ratio
DEGs: Differentially expressed genes
PANTHER: Protein analysis through evolutionary relationships
G2/M: Gap 2/mitosis
OS: Overall survival
DFS: Disease-free survival
SAC: Spindle assembly checkpoint
DNA: Deoxyribonucleic acid
APC/C: Anaphase promoting complex/cyclosome
